# Spatial Pattern and Determinants of Inadequate Complementary Feeding Indicators Among Children Aged 6–23 Months in Bangladesh: A Cross‐Sectional Study

**DOI:** 10.1002/hsr2.72241

**Published:** 2026-03-30

**Authors:** Satyajit Kundu, Shubhanjali Roy, Rakhi Dey, Saurav Basu, Mujibul Anam, Faruk Ahmed

**Affiliations:** ^1^ Public Health, School of Medicine and Dentistry Griffith University Gold Coast Queensland Australia; ^2^ ARCED Foundation Dhaka Bangladesh; ^3^ Indian Institute of Public Health‐Delhi, Public Health Foundation of India New Delhi India; ^4^ Statistics Discipline Khulna University Khulna Bangladesh; ^5^ Department of Community Medicine ESIC PGIMSR Medical College and Hospital Kolkata West Bengal India

**Keywords:** Bangladesh, complementary feeding, dietary diversity, infant and young child feeding, spatial analysis

## Abstract

**Background and Aims:**

Inadequate complementary feeding practices (CFPs) can lead to malnutrition among children and result in poor health outcomes throughout life. This study aimed to explore the spatial patterns and determinants of inadequate complementary feeding indicators (CFIs) among children aged 6–23 months in Bangladesh.

**Methods:**

We used data from the latest Bangladesh Demographic and Health Survey (BDHS) 2022. Three CFIs: minimum dietary diversity (MDD), minimum meal frequency (MMF), and minimum acceptable diet (MAD) were the outcome variables. Data from a weighted sample of 2578 children aged 6–23 months were included in this study. Hot spot analysis, ordinary Kriging interpolation, and multilevel binary logistic regression analyses were employed.

**Results:**

The overall prevalence of inadequate MDD, MMF, and MAD among children was 62.8%, 34.4%, and 70.4%, respectively. Spatial autocorrelation analysis showed that the distributions of inadequate MDD, MMF, and MAD across Bangladesh were highly clustered. For all three outcomes, a significant hot spot of inadequate feeding practice was noticed in the northeast region (Sylhet) of Bangladesh, while the cold spot was in the Southwest Khulna division. Young children (6–11 months), children of parents having no formal education, mothers with no employment, no media exposure, and belonging to households with low wealth status had higher odds of having inadequate MDD, MMF, and MAD. In contrast, children of mothers who had more frequent antenatal care (ANC) visits were less likely to have inadequate MMF and MAD.

**Conclusion:**

Socioeconomic and maternal factors, such as low parental education, lack of maternal employment, limited media exposure, and lower household wealth, were significantly associated with suboptimal CFPs. Monitoring appropriate CFIs nationwide and implementing policies and programs to address geographical and socioeconomic disparities are crucial for ensuring optimal child nutrition.

## 1 Introduction

Inadequate complementary feeding practices (CFPs) for children aged 6–23 months continue to pose a significant public health issue, especially in low‐ and middle‐income countries [1]. Inadequate CFPs refer to feeding practices that do not meet the recommended guidelines for introducing solid, semi‐solid, and soft foods to infants alongside continued breastfeeding [2]. In 2021, both the WHO and the United Nations Children's Fund (UNICEF) emphasized the importance of achieving key complementary feeding indicators (CFIs), such as minimum dietary diversity (MDD), minimum meal frequency (MMF), and minimum acceptable diet (MAD) among children [3]. The World Health Organization (WHO) defines MDD as the proportion of children who receive foods from at least five out of the eight recommended food groups over 24 h, while MMF refers to the minimum number of times a child should receive solid, semi‐solid, or soft foods in a day. MAD combines both MDD and MMF to evaluate whether children are receiving sufficient quantity and quality of food [4].

Adequate complementary feeding during the critical window of 6–23 months is essential for ensuring optimal physical growth, cognitive development, and immune function, while significantly reducing the risk of stunting, wasting, micronutrient deficiencies, and other malnutrition‐related illnesses [4, 5]. Globally, dietary diversity remains consistently low, with only about 29% of children meeting the MDD standard [6]. Among 80 countries analyzed, only 21.3%, 56.2%, and 10.1% achieved prevalence levels above 50% for MDD, MMF, and MAD, respectively. The lowest prevalence rates across all three indicators were observed in Western and Central Africa, while Latin America and the Caribbean reported the highest levels for MDD and MAD, and East Asia and the Pacific had the highest for MMF [1]. In the South Asian countries, such as India, Nepal, and Bangladesh, less than 40% of children receive a MAD [7]. The region bears one of the highest burdens of stunting globally, driven by inadequate CFPs [8].

Despite the adoption of the national policies aligned with the global guidelines, a significant proportion of children in Bangladesh continue to fail to meet these essential nutrition standards, thereby leading to a sustainable burden of malnutrition [9, 10]. Failure to meet essential CFIs, including MDD, MMF, and MAD, significantly exacerbates malnutrition, such as stunting and wasting, among children. Moreover, the lack of diversity in diets can lead to micronutrient deficiencies that have long‐term consequences on children's health [11].

Globally, nearly 45% of all deaths among children under five are due to undernutrition‐related conditions, such as stunting, wasting, and micronutrient deficiencies, primarily due to suboptimal CFPs [12]. This, in turn, impedes long‐term cognitive and physical development while heightening vulnerability to diseases [13]. Children who are malnourished due to poor CFPs are also more susceptible to frequent and severe infections, such as diarrheal diseases, respiratory tract infections, and measles, due to compromised immune function [14]. Moreover, the effects of undernutrition in early childhood can persist across the life course, contributing to reduced productivity in adulthood and increased risk of non‐communicable diseases such as diabetes and cardiovascular disorders [15].

The burden of malnutrition among children under five in Bangladesh is high, largely due to inadequate CFPs. According to the 2017 Bangladesh Demographic and Health Survey (BDHS), only 34% of children aged 6–23 months met the criteria for MAD, while only 26% achieved MDD [16]. Another study using the rural‐based nationwide survey in 2018–2019 reported that the prevalence of MAD was low at 18.3%, which is concerning given the equally low prevalence of MAD at 16.3% [17]. These statistics illustrate a widespread deficiency in both the quantity and variety of food consumed by children, contributing to high rates of stunting and underweight, which stand at 31% and 22%, respectively [16, 18]. Despite some progress in reducing malnutrition rates over the past two decades, such as a decline in stunting from 51% in 2004 to 31% in 2017, significant gaps remain [9]. A recent national survey indicates that approximately one in four children under 5 years old is stunted, primarily due to poor dietary practices and inadequate nutrition [19]. Alarmingly, the rate of wasting increased to 11% in Bangladeshi children under 5 years in 2022 [19] from 8% in 2017 [16], suggesting a deterioration in nutritional status among young children.

Studies indicate that socioeconomic factors, maternal education, maternal age, and geographic disparities significantly influence these practices, with children from poorer households, having mothers with no formal education, and rural areas being disproportionately affected [20, 21, 22, 23]. Maternal education has consistently been shown to positively impact infant and young child feeding practices by enhancing mothers' knowledge and decision‐making abilities [24]. Household wealth index is a key determinant of CFPs, as higher economic status improves access to diverse and nutritious foods and relevant information [25]. Maternal age also crucially determines the CFPs among children. For instance, adolescent motherhood plays a critical role in shaping infant nutrition, as young mothers often face challenges such as limited nutritional knowledge, inadequate economic resources, and insufficient social support, which can adversely impact CFPs [23]. Additionally, maternal health literacy is also a crucial determinant of infant feeding practices, as it influences a mother's ability to access, comprehend, and apply nutritional guidance, thereby directly impacting child nutrition outcomes [26]. Research highlights that regional variations in dietary intake significantly contribute to differences in malnutrition rates, as distinct food consumption patterns across regions can influence the availability and adequacy of essential nutrients, ultimately affecting nutritional outcomes [27].

Conducting a spatial analysis is vital as it allows researchers to visualize and understand the geographic distribution of inadequate feeding practices, thereby identifying hotspots that require urgent intervention. This approach can reveal how local conditions, such as poverty levels, access to resources, and educational opportunities, interact spatially to affect child nutrition outcomes [28, 29]. While a single study [30] initially explored the spatial pattern of CFPs using the BDHS 2014 data, their study was limited in scope and methodology. The previous study [30] only showed the relative density of CFIs, but did not perform hot spot analysis. In this study, we performed hot spot analysis to identify clusters of inadequate CFIs and used interpolation to predict prevalence in areas without direct data. Furthermore, regular assessments are essential to track current trends and patterns, addressing the need for up‐to‐date information. These enhancements provide a more comprehensive spatial understanding of CFPs in Bangladesh, facilitating more targeted interventions and policy development. Thus, this study seeks to fill critical gaps in knowledge and contribute to efforts aimed at improving child nutrition in Bangladesh, aligning with national health goals and global nutrition targets.

The objectives of this research were to estimate the prevalence and determinants of suboptimal CFIs among children aged 6–23 months in Bangladesh while also mapping the spatial pattern of inadequate MAD, MMF, and MDD across the country. The findings can support the enhancement of existing policy initiatives by guiding the development of region‐specific nutrition interventions and tailored maternal education initiatives that address context‐specific barriers, ultimately improving the CFIs among children aged 6–23 months in the most vulnerable areas.

## 2 Methods

### 2.1 Data Source, Study Design, and Participants

**FIGURE 1:**
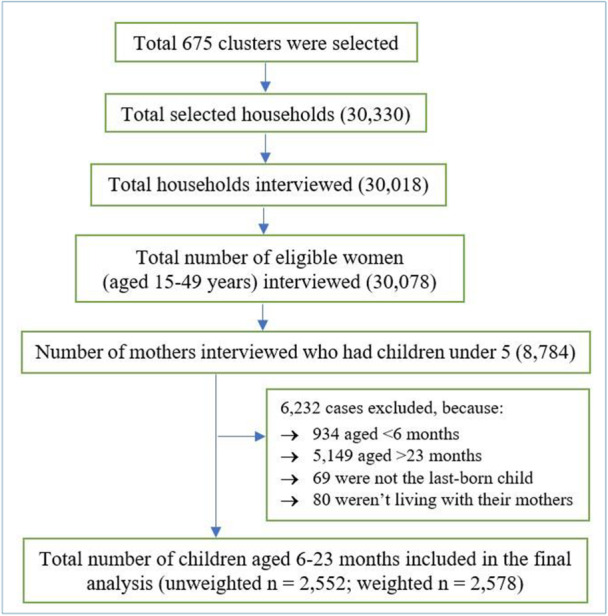
Flow chart of the participants' selection from Bangladesh Demographic and Health Survey (BDHS 2022) data.

This study used data from a nationally representative cross‐sectional survey of the BDHS 2022 [19]. In the BDHS 2022, a two‐stage stratified cluster sampling technique was adopted. In the first stage, 675 enumeration areas (EAs) were randomly selected from a complete list of EAs created for the 2011 population census in Bangladesh, with probability proportional to EA size [19]. In the second stage of sampling, an average of 45 households were chosen systematically from each EA and stratified for urban and rural areas from all eight divisions of Bangladesh [19]. A total of 30,078 ever‐married women aged 15–49 years were interviewed in this survey, including 8784 mothers of children aged 0–59 months [19]. The study included data on children aged 6–23 months only, who were the lastborn children of their mothers and were alive during the survey. The final weighted sample included in this study was 2578. A detailed sample selection procedure is provided in Figure 1.

### 2.2 Outcome Measures

Three CFIs, including MDD, MMF, and MAD, were the outcome variables. MDD was determined by the percentage of children who consumed foods and drinks from at least five out of eight specified food groups in the previous 24 h of the survey [1]. More information about these eight food groups is available in the BDHS 2022 final report [19]. MMF was assessed by the following criteria: breastfed infants aged 6–8 months needed two feedings of solid, semisolid, or soft foods; breastfed children aged 9–23 months required three feedings of these foods; and non‐breastfed children aged 6–23 months needed four feedings, which could include milk feeds, but at least one of the four must be a solid, semisolid, or soft food [1, 31]. Finally, MAD was determined by the percentage of children who met both the MDD and MMF criteria, and who were either breastfed or received at least two non‐human milk feeds in the 24 h before the survey [1]. All three outcome variables were binary, classified as “inadequate” vs. “adequate” based on joint WHO and UNICEF guidelines [32], with “inadequate” coded as 1 and “adequate” coded as 0.

### 2.3 Explanatory Variables

The explanatory variables included in this study were: age of mother (15–24 years, 25–34 years, ≥ 35 years), education of parents (no education, primary, secondary, and higher education), mothers' employment (yes and no), family size (≤ 5 members and > 5 members), sex of household head (male and female), religion (Muslim and non‐Muslim), household wealth status (poorest, poorer, middle, richer, and richest), media exposure (yes and no), antenatal care (ANC) visits (no visit, 1–3 visit, 4–7 visit, and ≥ 8 visits), place of delivery (home and institutional), parity (1–2 and ≥ 3), sex of the child (male and female), age of the child (6–11 months, 12–17 months, and 18–23 months), place of residence (urban and rural), and administrative divisions (Barisal, Chittagong, Dhaka, Khulna, Mymensingh, Rajshahi, Rangpur, and Sylhet).

The BDHS 2022 developed a wealth index using various household indicators, including asset ownership, housing materials, and access to utilities. A detailed description of household indicators used to calculate the wealth index can be found elsewhere [19]. Principal component analysis was applied to the standardized variables to calculate the wealth index. The index was then divided into five equal sections, known as quintiles, representing different wealth levels [33]. The media exposure variable was classified as “yes” if a woman reads a newspaper or magazine, watches television, or listens to the radio at least once a week. If she did not engage in any of these activities weekly, the variable was coded as “no” [21]. All the variables in the study were chosen based on prior research focusing on various CFPs, including MDD, MMF, and MAD [1, 18, 21, 22, 31, 34, 35, 36, 37, 38, 39].

### 2.4 Data Processing and Statistical Analysis

The data were cleaned, recoded, and analyzed with STATA version 17.0 (StataCorp, College Station, TX, USA). Initially, adjustments were made using sampling weights, primary sampling units, and strata using the “svy” command to enhance the survey's representativeness and the reliability of the findings. Descriptive statistics were then used to display frequencies and percentages, and the significance of bivariate analysis was estimated using the Chi‐square analysis. Spatial autocorrelation, hot spot analysis, and Kriging interpolation were conducted using ArcGIS version 10.8.

The weighted prevalence of MDD, MMF, and MAD was calculated for each cluster using STATA, and the prevalence was then combined with each cluster and geographic coordinates using ArcGIS software. Global spatial autocorrelation (Global Moran's I statistic) was used to examine the geographical distribution pattern of MDD, MMF, and MAD. Positive Moran's I values close to +1 indicate clustering, values near −1 suggest dispersion, and values around 0 imply random distribution of these feeding practices across the geographic area [39]. The Getis–Ord Gi* statistic was employed for hot spot analysis, utilizing *z*‐scores and significant *p*‐values. This analysis identified regions with notably high prevalence (hotspots) or low prevalence (cold spots) of inadequate MDD, MMF, and MAD among children aged 6–23 months throughout Bangladesh [39]. The spatial interpolation method was used to predict the prevalence range of inadequate MDD, MMF, and MAD among children in the unsampled areas based on point values from sampled clusters. For this, ordinary Kriging spatial interpolation was used, applying a spherical semivariogram model to interpolate the prevalence of inadequate MDD, MMF, and MAD based on observed point values in the unobserved areas of Bangladesh [38].

Since the BDHS 2022 used a two‐stage stratified cluster sampling method, creating a hierarchical data structure, a multilevel regression model was chosen as the best analytical approach to address and analyze cluster‐specific variations [40]. For this, we first fitted a null or intercept‐only model for outcome variables, keeping the cluster variable as a level‐2 factor without including any explanatory variables to test the cluster‐level variance. The significant cluster‐level variances (Supporting Information Table 1) in the null models justify the application of multilevel regression modeling in this study. Then, the final adjusted multilevel regression models, including all explanatory variables for all three outcomes, were employed to identify the determinants of inadequate MDD, MMF, and MAD. Although Chi‐square tests were initially used to explore bivariate associations, all explanatory variables, regardless of their significance in the Chi‐square tests, were included in the multilevel regression models to account for potential confounding effects. The findings were interpreted using the adjusted odds ratios (aORs) along with their 95% confidence intervals (CIs) from the adjusted regression models, keeping the significance level at *p* < 0.05. Before regression modeling, multicollinearity was assessed using the variance inflation factor (VIF), and no multicollinearity was detected. After applying multilevel models, the intra‐class correlation coefficient (ICC), proportionate change in variance (PCV), median odds ratio (MOR), deviance, and Akaike information criterion (AIC) were estimated as random‐effect estimates and model fitness for all three outcomes (Supporting Information Table 1).

### 2.5 Ethical Statement

The latest available BDHS 2022 dataset was used for this study. Registration was the only requirement for access to BDHS 2022 data. Therefore, ethical clearance was not required for this study. More details about DHS data and ethical standards are available online at (https://www.dhsprogram.com/).

## 3 Results

### 3.1 Background Characteristics of the Participants

Information from a total of 2578 (weighted) children aged 6–23 months was included in this study. Among the children, 50.3% were male, and 37.9% were aged 6–11 months. Most of the participants were from rural areas (73.7%), and the highest proportion was from the Dhaka division (23.9%), followed by Chattogram (21.8%), while the lowest percentage came from the Barishal division (5.9%). An equal distribution of nearly 20% of the participants was observed from each quintile of the household wealth index. Almost half of the mothers of children were aged 15–24 years, and about 5.4% of mothers did not have any formal education (Table 1).

**TABLE 1:** Background characteristics and distribution of prevalence (weighted) of inadequate minimum dietary diversity, meal frequency, and acceptable diet across variables.

Variables	Total; *n* (%)	Inadequate complementary feeding indicators (%)		
		MDD	MMF	MAD
Prevalence (95% CI)		62.8 (60.3–65.2)	34.4 (32.0–36.9)	70.4 (68.0–72.7)
Age of mother		*p* = 0.005	*p* = 0.318	*p* = 0.010
15–24 years	1264 (49.0)	66.3	36.0	73.4
25–34 years	1110 (43.1)	58.7	33.0	67.0
35 years or above	204 (7.9)	62.8	32.0	70.6
Education of mother		*p* < 0.001	*p* = 0.067	*p* < 0.001
No education	139 (5.4)	79.4	42.4	85.7
Primary	575 (22.3)	72.4	37.7	77.4
Secondary	1403 (54.4)	62.6	33.5	70.5
Higher	461 (17.9)	46.4	30.7	56.8
Education of father		*p* < 0.001	*p* < 0.001	*p* < 0.001
No education	420 (16.3)	73.6	42.2	80.3
Primary	763 (29.6)	68.4	34.5	73.9
Secondary	884 (34.3)	61.5	34.7	70.6
Higher	510 (19.8)	47.6	27.4	56.8
Mothers' working status		*p* = 0.087	*p* = 0.001	*p* = 0.016
No	1995 (77.4)	63.8	36.2	71.8
Yes	583 (22.6)	59.2	28.4	65.6
Family size		*p* = 0.518	*p* = 0.884	*p* = 0.729
≤ 5 members	1405 (54.5)	62.1	34.3	70.1
> 5 members	1173 (45.5)	63.6	34.6	70.8
Sex of household head		*p* = 0.811	*p* = 0.647	*p* = 0.535
Male	1290 (88.8)	62.9	34.2	70.1
Female	288 (11.2)	62.0	35.8	72.4
Religion		*p* = 0.502	*p* = 0.386	*p* = 0.360
Muslim	2391 (92.8)	63.0	34.7	70.7
Non‐Muslim	187 (7.24)	59.8	30.3	66.7
Wealth status		*p* < 0.001	*p* = 0.019	*p* < 0.001
Poorest	540 (21.0)	74.1	39.4	80.3
Poorer	547 (21.2)	66.6	37.3	73.0
Middle	527 (20.4)	65.8	32.7	72.6
Richer	501 (19.4)	59.0	33.5	67.0
Richest	464 (18.0)	45.8	28.2	57.1
Exposure to media		*p* < 0.001	*p* = 0.007	*p* < 0.001
No exposure	1382 (53.6)	67.1	37.0	74.1
Having exposure	1197 (46.4)	57.8	31.4	66.2
Number of ANC visits		*p* < 0.001	*p* = 0.001	*p* < 0.001
No visit	199 (7.7)	73.8	43.5	81.9
1–3 visits	1338 (51.9)	66.0	36.9	72.9
4–7 visits	916 (35.5)	57.1	29.6	65.2
8 visits or above	125 (4.9)	52.4	28.0	62.9
Place of delivery		*p* < 0.001	*p* = 0.015	*p* < 0.001
Home	938 (36.4)	68.0	37.8	75.2
Institutional	1640 (63.7)	59.8	32.5	67.7
Under 5 children in household		*p* = 0.001	*p* = 0.004	*p* = 0.002
0–1	1777 (68.9)	60.3	32.5	68.3
2 or above	801 (31.1)	68.2	38.7	75.1
Parity		*p* = 0.308	*p* = 0.643	*p* = 0.201
1–2	1919 (74.5)	62.1	34.1	69.7
3 or above	659 (25.6)	64.7	35.2	72.6
Birth order		*p* = 0.012	*p* = 0.288	*p* = 0.050
1	1003 (38.9)	60.5	32.9	69.2
2–3	1341 (52.0)	62.9	34.8	70.0
4 or above	235 (9.11)	71.9	38.8	77.9
Sex of child		*p* = 0.938	*p* = 0.494	*p* = 0.728
Male	1296 (50.3)	62.7	33.7	70.8
Female	1283 (49.8)	62.9	35.1	70.0
Age of child		*p* < 0.001	*p* < 0.001	*p* < 0.001
6–11 months	978 (37.9)	75.8	39.4	79.5
12–17 months	749 (29.1)	57.8	34.2	66.9
18–23 months	851 (33.0)	52.2	28.9	63.0
Place of residence		*p* = 0.012	*p* = 0.009	*p* = 0.011
Urban	678 (26.3)	57.3	29.1	65.4
Rural	1900 (73.7)	64.7	36.3	72.2
Administrative divisions		*p* < 0.001	*p* < 0.001	*p* < 0.001
Barisal	153 (5.9)	71.4	44.9	77.7
Chittagong	562 (21.8)	72.1	48.7	79.7
Dhaka	616 (23.9)	57.3	28.4	64.8
Khulna	265 (10.3)	47.5	18.2	52.5
Mymensingh	237 (9.2)	56.0	33.5	66.4
Rajshahi	263 (10.2)	63.5	30.9	70.8
Rangpur	304 (11.8)	63.9	28.1	73.5
Sylhet	178 (6.9)	73.9	42.3	80.1

*Note:* All the percentages presented in the table are calculated by row. The *p*‐values < 0.005 were considered statistically significant.

Abbreviations: ANC, antenatal care; CI, confidence interval; MAD, minimum acceptable diet; MDD, minimum dietary diversity; MMF, minimum meal frequency. *p* values were calculated using the Chi‐square test.

### 3.2 Distribution of Prevalence of MDD, MMF, and MAD Across Variables

The overall prevalence of inadequate MDD, MMF, and MAD among children was 62.8 (95% CI: 60.3–65.2), 34.4 (95% CI: 32.0–36.9), and 70.4 (95% CI: 68.0–72.7), respectively. Children of younger mothers aged 15–24 years had the highest prevalence of inadequate MDD (66.3%), MMF (36.0%), and MAD (73.4%) compared to children of older mothers. Similarly, children of mothers with no formal education showed the highest prevalence of inadequate MDD (79.4%), MMF (42.4%), and MAD (85.7%) compared to children of mothers having higher education. A higher prevalence of inadequate MDD (74.1%), MMF (39.4%), and MAD (80.3%) was found among children from the poorest wealth quintile compared to the other quintiles. Compared to the older children, who were younger, aged 6–11 months, had a higher prevalence of inadequate MDD (75.8%), MMF (39.4%), and MAD (79.5%). Children of mothers who had no ANC visit, no media exposure, and were delivered at home were found to have a higher prevalence of inadequate MDD, MMF, and MAD. According to the bivariate analysis, the prevalence of inadequate MDD, MMF, and MAD did not vary significantly by family size, sex of household head, religion, parity, and sex of child (Table 1).

### 3.3 Spatial Pattern of MDD, MMF, and MAD Among Children in Bangladesh

**FIGURE 2:**
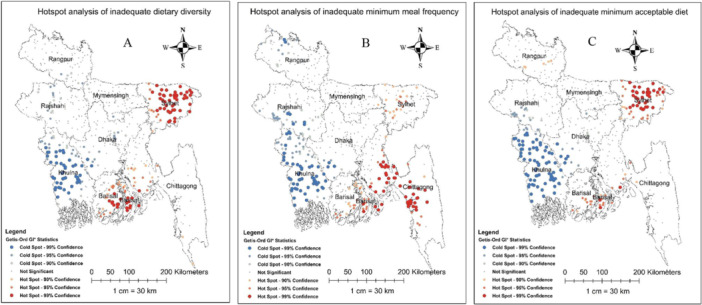
(A) Shows the spatial clustering (hot spot and cold spot) of inadequate MDD, (B) shows the spatial clustering (hot spot and cold spot) of inadequate MMF, and (C) shows the spatial clustering (hot spot and cold spot) of inadequate MAD among children in Bangladesh (maps were generated using ArcGIS v 10.8 software).

The global spatial autocorrelation analysis showed that the distributions of inadequate MDD (global Moran's I = 0.096, *p* < 0.001), MMF (I = 0.065, *p* < 0.001), and MAD (I = 0.093, *p* < 0.001) among children across Bangladesh were clustered (Supporting Information Figures 1–3). The hot spots for higher prevalence of inadequate MDD were identified in the Sylhet and Barisal divisions (Figure 2A), while the hot spots for higher prevalence of inadequate MMF were identified in the Barisal and Chittagong divisions (Figure 2B). Similarly to the MDD, the hot spots for higher prevalence of inadequate MAD were identified in the Sylhet division (Figure 2C).

The highest predicted prevalence (ranging from 72% to 92%) of inadequate MDD was mostly observed in the northern part of Sylhet and the southern parts of the Barisal and Chittagong divisions (Supporting Information Figure 4). Similarly, the highest predicted prevalence of inadequate MMF was found in the southern part of Barisal, the northern part of Sylhet and most of the Chittagong division (Supporting Information Figure 5), and the highest predicted prevalence (ranging from 79% to 96%) of inadequate MAD was mostly observed in most of Sylhet, the southern parts of the Barisal and Chittagong, and the eastern part of the Rangpur division (Supporting Information Figure 6). While both cold spots (lowest prevalence) and the lowest predicted prevalence of inadequate CFIs (MDD, MMF, and MAD) were identified in the Khulna division.

### 3.4 Factors Associated With Inadequate MDD

After adjustment, the likelihood of having inadequate MDD and its associated factors was as follows. Children having parents with no formal education or only primary education were more likely to have inadequate MDD compared to those having parents with higher education. Compared to the richest wealth quintile, children from other wealth groups had higher odds of having inadequate MDD. For example, the poorest quintile showed 1.99 times higher odds of having inadequate MDD than the richest quintile (AOR = 1.99; 95% CI: 1.35, 2.92). Children of mothers having no media exposure [aOR 1.31 (1.07, 1.60)], and whose birth order was between 2 and 3 (AOR = 1.32; 95% CI: 1.02, 1.71) had higher odds of having inadequate MDD compared to their counterparts. Younger children aged 6–11 months (AOR = 3.22; 95% CI: 2.56, 4.03) and 12–17 months (AOR = 1.31; 95% CI: 1.04, 1.63) had higher odds of having inadequate MDD compared to children aged 18–23 months (Table 2).

**TABLE 2:** Determinants of inadequate minimum dietary diversity, meal frequency, and acceptable diet among children aged 6–23 months in Bangladesh.

Variables	Inadequate complementary feeding indicators; aOR (95% CI)		
	MDD	MMF	MAD
Age of mother			
15–24 years (ref)	Reference	Reference	Reference
25–34 years	0.76 (0.59–0.98)*	0.91 (0.72–1.63)	0.83 (0.64–1.08)
35 years or above	0.82 (0.53–1.27)	0.88 (0.58–1.35)	0.83 (0.53–1.30)
Education of mother			
No education	2.09 (1.18–3.70)*	0.99 (0.61–1.63)	2.00 (1.07–3.74)*
Primary	1.77 (1.23–2.54)**	0.75 (0.53–1.06)	1.39 (0.96–2.02)
Secondary	1.18 (0.89–1.57)	0.77 (0.58–1.03)	1.11 (0.83–1.48)
Higher (ref)	Reference	Reference	Reference
Education of father			
No education	1.62 (1.11–2.37)*	1.56 (1.08–2.25)*	1.91 (1.29–2.84)**
Primary	1.39 (1.02–1.91)*	1.24 (0.90–1.70)	1.36 (0.99–1.87)
Secondary	1.29 (0.98–1.70)	1.25 (0.94–1.67)	1.41 (1.07–1.86)*
Higher (ref)	Reference	Reference	Reference
Mothers' working status			
No	1.24 (0.99–1.55)	1.37 (1.09–1.73)**	1.38 (1.09–1.73)**
Yes (ref)	Reference	Reference	Reference
Family size			
≤ 5 members (ref)	Reference	Reference	Reference
> 5 members	1.01 (0.83–1.23)	0.99 (0.74–1.07)	0.97 (0.80–1.19)
Sex of household head			
Male (ref)	Reference	Reference	Reference
Female	1.07 (0.79–1.44)	0.94 (0.71–1.26)	1.25 (0.91–1.71)
Religion			
Muslim (ref)	Reference	Reference	Reference
Non‐Muslim	0.86 (0.61–1.22)	0.78 (0.55–1.11)	0.83 (0.59–1.18)
Wealth status			
Poorest	1.99 (1.35–2.92)**	1.07 (0.74–1.56)	1.71 (1.15–2.54)**
Poorer	1.69 (1.19–2.39)**	1.11 (0.79–1.56)	1.42 (1.00–2.02)*
Middle	1.61 (1.17–2.22)**	0.91 (0.66–1.26)	1.39 (1.01–1.93)*
Richer	1.36 (1.01–1.84)*	1.06 (0.78–1.44)	1.25 (0.93–1.70)
Richest (ref)	Reference	Reference	Reference
Exposure to media			
No exposure	1.31 (1.07–1.60)**	1.18 (0.97–1.44)	1.30 (1.06–1.60)*
Having exposure (ref)	Reference	Reference	Reference
Number of ANC visits			
No visit (ref)	Reference	Reference	Reference
1–3 visits	0.88 (0.60–1.29)	0.74 (0.53–1.03)	0.65 (0.42–0.99)*
4–7 visits	0.77 (0.51–1.15)	0.61 (0.42–0.87)**	0.59 (0.37–0.92)*
8 visits or above	0.85 (0.49–1.51)	0.51 (0.30–0.89)*	0.62 (0.34–1.12)
Place of delivery			
Home (ref)	Reference	Reference	Reference
Institutional	0.99 (0.78–1.23)	0.97 (0.79–1.11)	1.04 (0.83–1.30)
Under 5 children in household			
0–1 (ref)	Reference	Reference	Reference
2 or above	1.08 (0.71–1.25)	1.03 (0.83–1.28)	1.00 (0.80–1.27)
Parity			
1–2 (ref)	Reference	Reference	Reference
3 or above	0.82 (0.61–1.10)	0.85 (0.64–1.12)	0.86 (0.64–1.16)
Birth order			
1 (ref)	Reference	Reference	Reference
2–3	1.32 (1.02–1.71)*	1.19 (0.93–1.53)	1.75 (0.90–1.53)
4 or above	1.42 (0.87–1.32)	1.26 (0.79–2.01)	1.10 (0.66–1.83)
Sex of child			
Male (ref)	Reference	Reference	Reference
Female	0.97 (0.81–1.17)	0.99 (0.83–1.18)	0.94 (0.78–1.50)
Age of child			
6–11 months	3.22 (2.56–4.03)***	1.52 (1.23–1.87)***	2.33 (1.85–2.94)***
12–17 months	1.31 (1.04–1.63)*	1.23 (0.98–1.55)	1.19 (0.78–1.13)
18–23 months (ref)	Reference	Reference	Reference
Place of residence			
Urban	1.04 (0.83–1.31)	0.90 (0.72–1.13)	1.01 (0.81–1.27)
Rural (ref)	Reference	Reference	Reference
Administrative divisions			
Barisal	1.52 (1.02–2.29)*	1.47 (1.01–2.14)*	1.53 (1.02–2.31)*
Chittagong	1.68 (1.17–2.41)**	2.13 (1.53–2.97)***	1.91 (1.33–2.75)**
Dhaka (ref)	Reference	Reference	Reference
Khulna	0.65 (0.45–0.95)*	0.53 (0.36–0.80)**	0.59 (0.41–0.85)**
Mymensingh	0.78 (0.54–1.14)	1.06 (0.73–1.53)	0.91 (0.62–1.31)
Rajshahi	1.20 (0.81–1.79)	1.00 (0.68–1.48)	1.24 (0.83–1.83)
Rangpur	1.11 (0.76–1.64)	0.89 (0.61–1.31)	1.38 (0.94–2.04)
Sylhet	1.86 (1.25–2.79)**	1.49 (1.03–2.14)*	1.82 (1.21–2.74)**

Abbreviations: ANC, antenatal care; aOR, adjusted odds ratio; CI, confidence interval; MAD, minimum acceptable diet; MDD, minimum dietary diversity; MMF, minimum meal frequency; ref, reference.

* *p*‐value < 0.05

** *p*‐value < 0.01

*** *p*‐value < 0.001.

### 3.5 Factors Associated With Inadequate MMF

Children of fathers having no formal education (AOR = 1.56; 95% CI: 1.08, 2.25) and mothers having no working status (AOR = 1.37; 95% CI: 1.09, 1.73) were more likely to get inadequate MMF compared to those who had fathers with higher education and mothers involved in work. Children aged 6–11 months had 52% higher odds of having inadequate MMF compared to children aged 18–23 months (AOR = 1.52; 95% CI: 1.23, 1.87). In the case of ANC, children of mothers who had 4–7 ANC visits (AOR = 0.61; 95% CI: 0.42, 0.87) or ≥ 8 ANC visits (AOR = 0.51; 95% CI: 0.30, 0.89) were less likely to get inadequate MMF compared to those who didn't have any ANC (Table 2).

### 3.6 Factors Associated With Inadequate MAD

Children with parents who had less education were more likely to have inadequate MAD compared to those with parents who had higher education. For example, mothers with no education and fathers with no education were found to have 2.00 times (AOR = 2.00; 95% CI: 1.07, 3.74) and 1.91 times (AOR = 1.91; 95% CI: 1.29, 2.84) higher odds of getting inadequate MAD, respectively, compared to those having higher education. Compared to the richest wealth quintile, children from other wealth quintiles had higher odds of having inadequate MAD. For example, the poorest quintile showed 1.71 times higher odds of having inadequate MAD than the richest quintile (AOR = 1.71; 95% CI: 1.15, 2.54). Children of mothers having no work involvement [aOR 1.38 (1.09, 1.73)], and no media exposure (AOR = 1.30; 95% CI: 1.06, 1.60) had higher odds of having inadequate MAD compared to their counterparts. When looking at the ANC visits of mothers, children of mothers who had 1–3 visits (AOR = 0.65; 95% CI: 0.42, 0.99) or 4–7 visits (AOR = 0.59; 95% CI: 0.37, 0.92) were less likely to get inadequate MAD compared to those who didn't have any ANC visit. Younger children aged 6–11 months had 2.33 times higher odds of having inadequate MAD compared to children aged 18–23 months (AOR = 2.33; 95% CI: 1.85, 2.94) (Table 2).

## 4 Discussion

The findings from this study highlight significant disparities in CFPs by revealing the geospatial patterns and key determinants of inadequate CFIs among children aged 6–23 months in Bangladesh. This study presents a comprehensive overview of trends, spatial clustering, and the maternal and socioeconomic factors associated with CFPs.

The prevalence of inadequate CFPs among children aged 6–23 months in Bangladesh has shown mixed trends over the past decade. According to BDHS 2011 and 2022, the rate of inadequate MDD has decreased from 76% in 2011 to 62.8% in 2022 [19, 41], and the progress in other indicators has stagnated or even reversed. The prevalence of inadequate MMF has worsened, increasing from 19% in 2017–2018 to 34.4% in 2022. Similarly, the prevalence rate of inadequate MAD has risen from 65% in 2017–2018 to 70.4% in 2022 [16]. These diverging trends suggest that while some progress has been made in improving dietary diversity, other aspects of feeding, such as frequency and overall adequacy, may have deteriorated due to socioeconomic strain, knowledge gaps, or a lack of comprehensive implementation of feeding programs. This increase in inadequate MAD is particularly concerning as it falls far short of the government's target of reducing it to 55% by 2022, as outlined in the Fourth Health, Population and Nutrition Sector Program (HPNSP) in the years 2017–2022 [41]. This target, established under the 2017–2022 implementation cycle of the HPNSP, was likely unmet due to a combination of policy and programmatic shortcomings, further exacerbated by external shocks, including the COVID‐19 pandemic, macroeconomic disruptions, and enduring systemic weaknesses within the health infrastructure [10]. These trends highlight a significant gap between policy goals and implementation, underscoring the need for more effective interventions to improve CFPs among children in Bangladesh. Several key determinants that were significantly associated with feeding practices included the child's age and gender, parental education, ANC visits, and household wealth quintile.

Children of younger mothers (aged 15–24 years) had the highest prevalence of inadequate MDD (66.3%), MMF (36.0%), and MAD (73.4%) compared to children of mothers aged 25–34 years and 35 years and above. Younger mothers may be more likely to have limited experience with child feeding practices and reduced access to health and nutrition information, which could influence the quality and frequency of complementary foods offered to their children [42, 43]. Additionally, younger mothers often find themselves in lower socioeconomic positions, which can restrict their access to resources necessary for providing adequate nutrition. Economic constraints may force them to rely on cheaper, less nutritious food options, resulting in insufficient dietary diversity and frequency for their children [44]. This aligns with existing literature indicating that maternal age is a significant determinant of child nutrition outcomes, where younger mothers often face challenges related to education, socioeconomic status, and access to health services [45].

Parental education plays a significant role in shaping feeding practices, particularly in the context of infant and young child feeding practices. Research indicates that higher levels of parental education are generally associated with better feeding practices, such as increased dietary diversity and adherence to recommended meal frequencies. For instance, studies have shown that educated parents are more likely to introduce a variety of foods and ensure that their children receive a MAD [46, 47]. Mothers exposed to nutrition‐focused media campaigns or health messaging are more likely to understand the importance of dietary diversity and feeding frequency, and apply this knowledge in practice [48]. Our findings support this positive association. However, studies from some rural or low‐literacy settings have noted a limited impact of media exposure, especially when access to mass media is constrained, or content is not culturally adapted [49]. These contrasting findings highlight the importance of tailoring communication strategies to local contexts to ensure sustainable behavioral change.

The analysis revealed that socioeconomic status is a critical determinant of CFPs. Children from poorer wealth quintiles showed significantly higher odds of inadequate MDD, MMF, and MAD compared to those from wealthier families. For example, children in the poorest quintile had nearly double the odds of inadequate MDD compared to those in the richest quintile. This finding corroborates existing literature that highlights the detrimental impact of poverty on child nutrition [9, 50]. Economic constraints often limit access to diverse food sources, leading to suboptimal dietary practices among vulnerable populations. Moreover, families with lower socioeconomic status may prioritize quantity over quality in their children's diets due to financial limitations, resulting in a lack of essential nutrients necessary for healthy growth [30, 51]. This underscores the importance of addressing economic disparities through social protection programs that enhance food security and promote access to nutritious foods for low‐income families.

The association between ANC visits and CFPs is particularly noteworthy. Children whose mothers had more ANC visits were less likely to exhibit inadequate MMF and MAD compared to their counterparts. This finding emphasizes the importance of maternal health services in promoting better feeding practices and aligns with previous research demonstrating that ANC can enhance maternal knowledge about child nutrition [52]. Furthermore, mothers who received adequate ANC are more likely to be informed about best practices for infant feeding and child care, which can translate into improved dietary diversity for their children [53]. The study's results suggest that enhancing access to quality ANC services could play a pivotal role in improving CFPs across Bangladesh.

Additionally, the lack of media exposure among mothers was associated with higher odds of inadequate feeding indicators, suggesting that access to information plays a crucial role in shaping dietary behaviors. Research indicates that mothers who are less exposed to media campaigns often lack awareness of optimal CFPs, which can lead to poor nutritional outcomes for their children. For instance, studies have shown that targeted media campaigns can effectively educate parents about the importance of diverse diets and proper meal frequencies, thereby improving child nutrition outcomes [54]. Media campaigns aimed at educating parents about optimal CFPs could be an effective strategy for improving child nutrition outcomes [55].

Younger children aged 6–11 months displayed a higher likelihood of inadequate MDD, MMF, and MAD compared to older children aged 18–23 months. This age group is particularly vulnerable as they transition from exclusive breastfeeding to complementary foods, making it essential to ensure that their dietary needs are met during this critical period. The nutritional needs of infants in the 6‐ to 11‐month age range are critical as they transition from exclusive breastfeeding to complementary feeding, which requires careful attention to dietary diversity and frequency to ensure adequate nutrient intake. Research indicates that children in this younger age group are 75% less likely to meet MDD compared to their older counterparts, primarily due to the limited variety of foods introduced during this crucial developmental phase [17]. Additionally, younger children often have less exposure to a variety of foods, as caregivers may be hesitant to introduce new foods or may lack knowledge about appropriate CFPs. This lack of exposure can lead to a reliance on a narrow range of foods, typically cereals and breast milk, which do not meet the diverse nutritional needs of growing infants [22]. The findings suggest a need for targeted educational interventions aimed at caregivers of younger children to improve their understanding of appropriate CFPs [56].

The spatial analysis identified hot spots for inadequate MDD, MMF, and MAD primarily in the Sylhet and Barisal divisions. The global spatial autocorrelation results indicate that these inadequacies are not randomly distributed but rather clustered in specific regions, suggesting that localized interventions may be necessary to address these disparities effectively. These findings are consistent with other regional reports that have also shown poorer child CFPs and nutrition outcomes in these divisions [57, 58].

The high prevalence rates observed in these regions may be attributed to several factors, including limited access to healthcare services, lower levels of maternal education, cultural beliefs surrounding feeding practices, and regional agricultural conditions that affect food availability. For instance, the Sylhet division is characterized by higher poverty rates, lower maternal literacy, and limited ANC coverage compared to other regions [16]. In Barisal, geographic remoteness and seasonal flooding reduce access to markets, affecting the year‐round availability of diverse foods [59, 60]. Targeted interventions in these hot spot areas could involve community‐based nutrition education programs that empower caregivers with knowledge about appropriate CFPs while considering local cultural contexts. In addition to education, we recommend tailored interventions such as mobile nutrition counseling units, region‐specific SBCC campaigns, and integration of nutrition‐sensitive agriculture and food assistance schemes in flood‐prone zones.

### 4.1 Strengths and Limitations

This study has several strengths that enhance its reliability and applicability. First, the large sample size with country‐level representativeness provides a robust foundation for generalizability across various demographic groups, facilitating a more accurate representation of CFPs in Bangladesh. Additionally, the incorporation of geospatial analysis offers valuable insights into regional disparities, enabling targeted interventions based on geographic needs. The multilevel approach employed in this study takes into account individual, household, and community‐level factors, offering a comprehensive understanding of the determinants that affect CFPs. However, there are several study limitations too. The cross‐sectional design limits the ability to establish causal relationships between variables, highlighting the need for longitudinal studies for a more in‐depth understanding. Additionally, the reliance on self‐reported data may introduce biases or inaccuracies regarding dietary practices, which could impact the validity of the findings. To minimize this, the analysis used standardized 24‐h recall questions consistent with DHS protocols; however, residual bias cannot be fully excluded. Future studies could incorporate objective measures, repeated recall, or validation subsamples to improve dietary accuracy. Also, while the study identifies key determinants of inadequate CFPs, it did not allow us to explore cultural beliefs or practices, due to a lack of data, that may also influence these behaviors, which could provide further context for interpreting the observed findings. Future research should explore the role of cultural beliefs and social norms in shaping CFP‐related behaviors to provide a more holistic understanding. Another limitation is that the MAD indicator used in this study does not account for the quantitative nutrient adequacy of complementary foods, nor does it assess the quality of the diet in terms of micronutrient content or diet‐related risks such as childhood overweight and obesity [61].

### 4.2 Recommendations and Policy Implications

The present study indicates that nearly two‐thirds of Bangladeshi children aged 6–23 months had inadequate MAD and MDD, while one in three children had inadequate MMF, indicating meals that were deficient in key nutritional requirements. The implementation of targeted supplementary feeding programs, such as the integrated child development scheme in India [62] for infants beyond 6 months of age and young children could be considered to improve the dietary intake and nutritional status of this vulnerable cohort. There was some evidence that children under 2 years of age and poorly nourished children benefited in terms of improved weight and height through supplementary feeding [63]. Nutrition social behavior change communication interventions in primary care and community‐based settings targeting young mothers and child caregivers toward improving their children's diet should be prioritized, as there was evidence of mothers with lower education having poorer child feeding practices contributing to a higher burden of inadequate MDD [64].

Specifically, peer counseling is likely to be a cost‐effective strategy to improve CFPs, including initiation and attainment of MMF and MAD in Bangladesh [65, 66]. Such interventions could be designed and implemented as site‐specific programs, particularly in areas where significant hot spots of lower prevalence of CFIs were identified. Children whose mothers received ANC were less likely to have reduced MMF and MAD, possibly due to nutritional counseling during pregnancy. So, improving the quality and accessibility of nutritional counseling during ANC could further enhance complementary feeding among young children. Capacity building of frontline workers and increasing the duration, frequency, and content of counseling sessions is urgently warranted in this regard [67].

## 5 Conclusion

Inadequate CFPs remained a critical public health concern in Bangladesh, with particularly high prevalence of inadequate MAD (70.4%) and notable geographic clustering of poor feeding indicators in the Sylhet region. Socioeconomic and maternal factors, such as low parental education, lack of maternal employment, limited media exposure, and lower household wealth, were significantly associated with suboptimal feeding practices. We found that the overall status of complementary feeding was suboptimal, falling behind the target outlined in the Fourth Health, Population and Nutrition Sector Program (HPNSP) by the government of Bangladesh. This highlights the need for urgent action through integrated, multisectoral interventions that address not only nutritional education but also socioeconomic barriers that hinder access to diverse food sources. Future policies should prioritize enhancing maternal education and increasing access to health services, particularly antenatal care and nutrition counseling. Moreover, addressing the challenges surrounding CFPs is essential for reducing malnutrition and promoting healthy growth among young children in Bangladesh. Ongoing monitoring and evaluation of feeding practices, combined with targeted interventions, will be essential for maintaining progress and ensuring that all children receive the necessary nutrition during this vital developmental stage.

## Author Contributions

**Satyajit Kundu:** conceptualization, methodology, software, data curation, investigation, formal analysis, writing – review and editing, writing – original draft. **Shubhanjali Roy:** writing – original draft, writing – review and editing. **Rakhi Dey:** validation, writing – original draft, writing – review and editing, data curation. **Saurav Basu:** visualization, writing – review and editing, writing – original draft. **Faruk Ahmed:** investigation, supervision, validation, visualization, writing – review and editing.

## Conflicts of Interest

The authors declare no conflicts of interest.

## Transparency Statement

The lead author Satyajit Kundu affirms that this manuscript is an honest, accurate, and transparent account of the study being reported; that no important aspects of the study have been omitted; and that any discrepancies from the study as planned (and, if relevant, registered) have been explained.

## Supporting information

Supplementary File.

## Data Availability

The data that support the findings of this study are available on request from the corresponding author. The data are not publicly available due to privacy or ethical restrictions.
