# Supplementary material for: Spatial Pattern and Determinants of Inadequate Complementary Feeding Indicators Among Children Aged 6–23 Months in Bangladesh: A Cross‐Sectional Study
Source: Health Sci Rep. 2026 Mar 30;9(4):e72241. doi: 10.1002/hsr2.72241 (PMC13087518; doi:10.1002/hsr2.72241)
Supplement: Supplementary file 1 — Supplementary File. [file HSR2-9-e72241-s001.docx]

**Supplementary Table 1.** Random effect estimates and model fitness of multilevel models

| **Parameters** | **Null or intercept-only model** | **Final adjusted model** |
| --- | --- | --- |
| **Minimum dietary diversity** |  |  |
| Variance (95% CI) | 0.46 (0.29 – 0.74) | 0.23 (0.10 – 0.53) |
| ICC (%) | 12.36 | 6.63 |
| PCV (%) | Reference | 50.00 |
| MOR | 1.90 | 1.58 |
| Deviance | 3347.00 | 3016.47 |
| AIC | 3351.00 | 3092.47 |
| **Minimum meal frequency** |  |  |
| Variance (95% CI) | 0.38 (0.23 – 0.64) | 0.15 (0.05 – 0.44) |
| ICC (%) | 10.44 | 4.26 |
| PCV (%) | Reference | 60.53 |
| MOR | 1.80 | 1.44 |
| Deviance | 3259.42 | 3117.20 |
| AIC | 3263.42 | 3193.19 |
| **Minimum acceptable diet** |  |  |
| Variance (95% CI) | 0.46 (0.28 – 0.75) | 0.17 (0.06 – 0.50) |
| ICC (%) | 12.19 | 4.87 |
| PCV (%) | Reference | 63.04 |
| MOR | 1.90 | 1.48 |
| Deviance | 3080.93 | 2834.96 |
| AIC | 3084.93 | 2910.96 |
| CI: Confidence interval, ICC: Intraclass correlation, PCV: Proportional change in variance, MOR: Median odds ratio, AIC: Akaike information criterion | | |


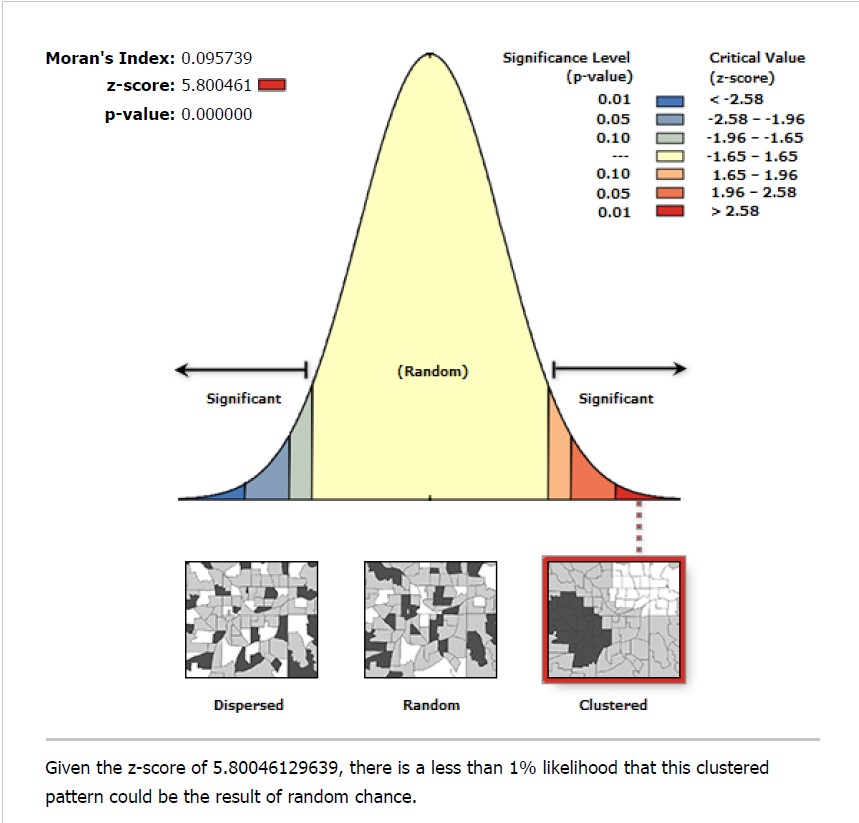


**Supplementary Figure 1**. Global spatial autocorrelation report showing the inadequate MDD among children in Bangladesh


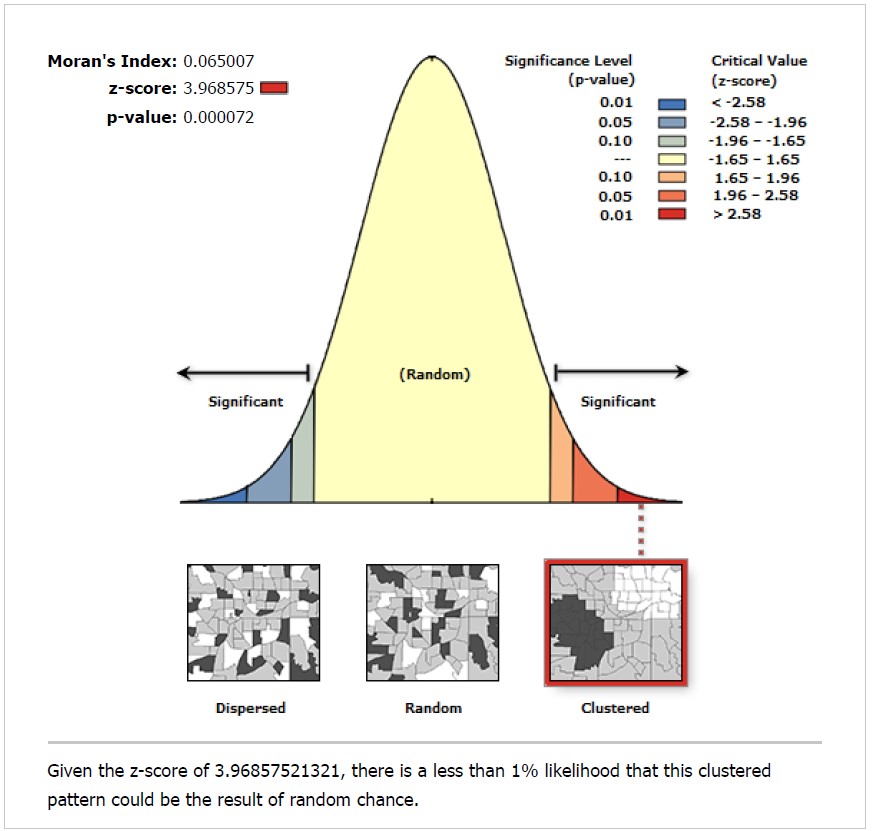


**Supplementary Figure 2**. Global spatial autocorrelation report showing the inadequate MMF among children in Bangladesh


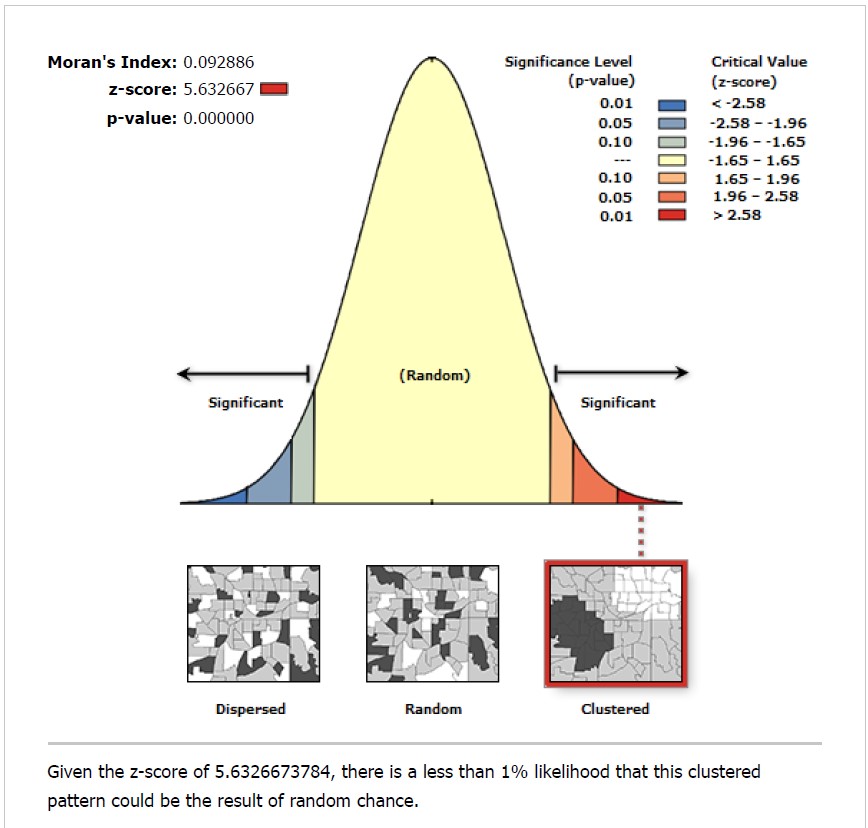


**Supplementary Figure 3**. Global spatial autocorrelation report showing the inadequate MAD among children in Bangladesh


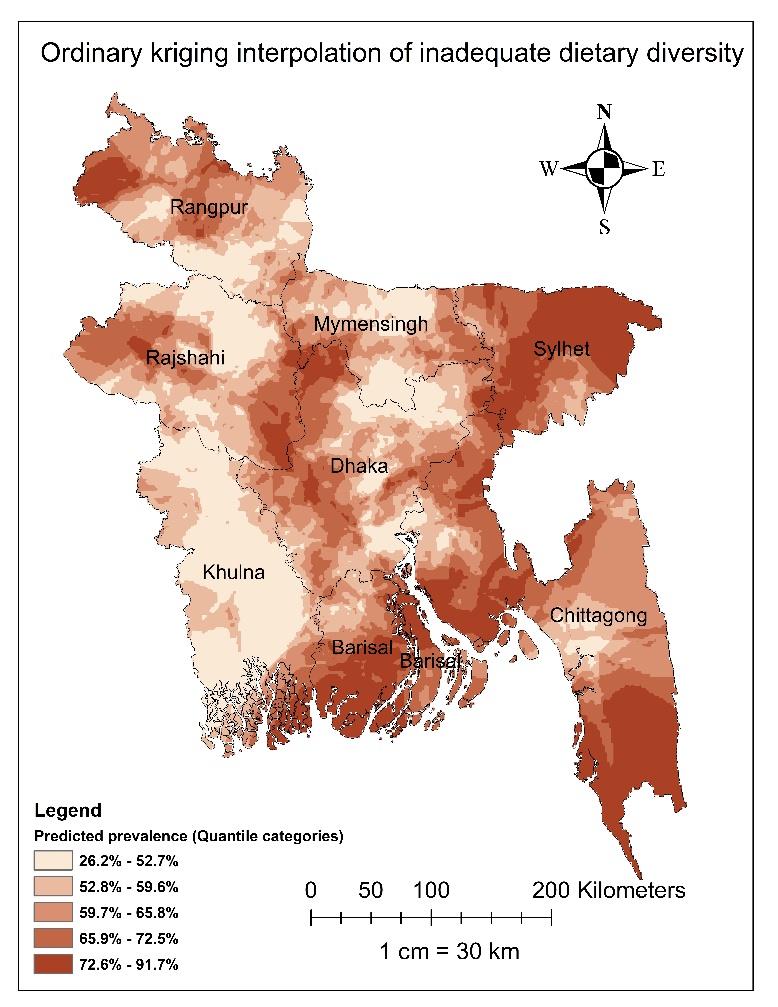


**Supplementary Figure 4**. Spatial interpolation of inadequate MDD among children in Bangladesh (maps were generated using ArcGIS v 10.8 software)


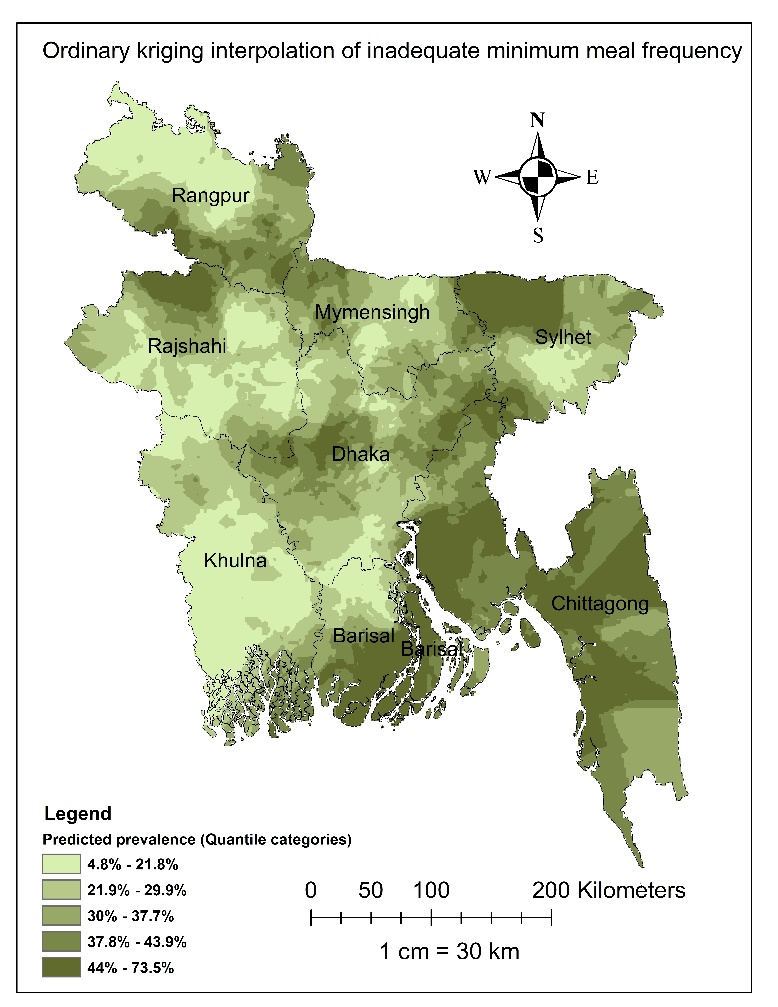


**Supplementary Figure 5**. Spatial interpolation of inadequate MMF among children in Bangladesh (maps were generated using ArcGIS v 10.8 software)

**
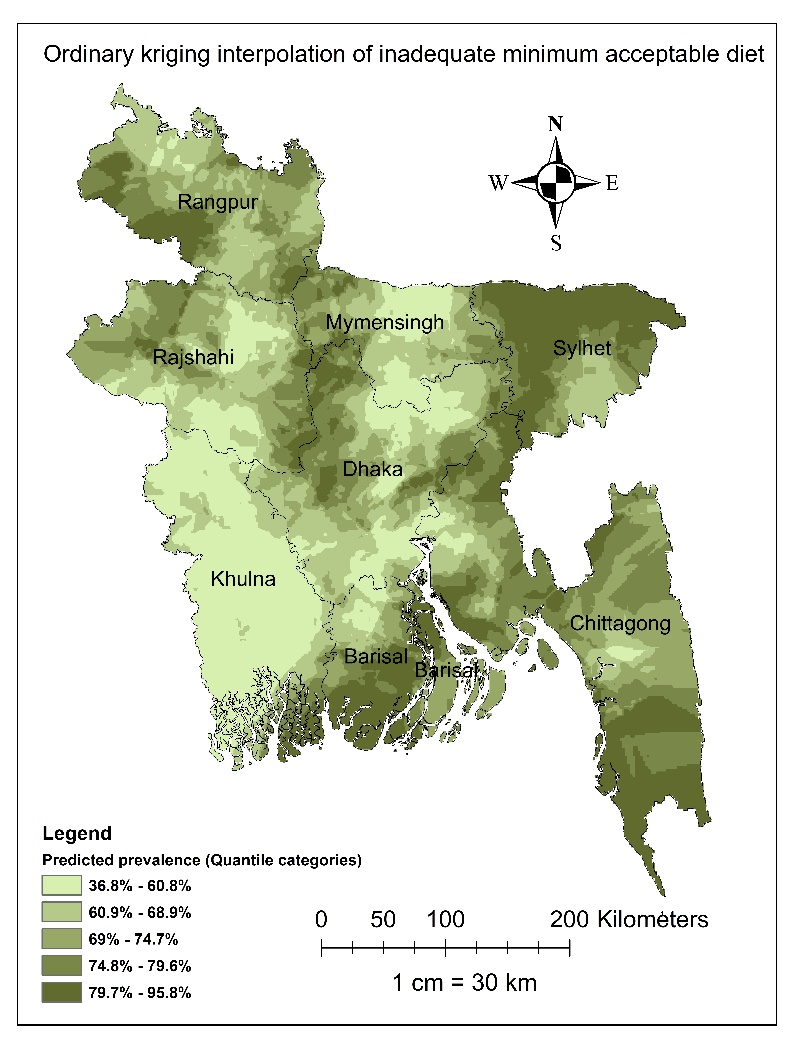
**

**Supplementary Figure 6**. Spatial interpolation of inadequate MAD among children in Bangladesh (maps were generated using ArcGIS v 10.8 software)
